# An updated, computable MEDication-Indication resource for biomedical research

**DOI:** 10.1038/s41598-021-98579-4

**Published:** 2021-09-23

**Authors:** Neil S. Zheng, V. Eric Kerchberger, Victor A. Borza, H. Nur Eken, Joshua C. Smith, Wei-Qi Wei

**Affiliations:** 1 Department of Biomedical Informatics, Vanderbilt University Medical Center, Nashville, TN USA; 2 Yale School of Medicine, New Haven, CT USA; 3 Division of Allergy, Pulmonary and Critical Care Medicine, Department of Medicine, Vanderbilt University Medical Center, Nashville, TN USA; 4 Vanderbilt School of Medicine, Nashville, TN USA; 5 Department of Biomedical Informatics, Vanderbilt University Medical Center, 2525 West End Avenue Suite 1500, Nashville, TN 37232-6602 USA

**Keywords:** Data publication and archiving, Medical research, Drug therapy

## Abstract

The MEDication-Indication (MEDI) knowledgebase has been utilized in research with electronic health records (EHRs) since its publication in 2013. To account for new drugs and terminology updates, we rebuilt MEDI to overhaul the knowledgebase for modern EHRs. Indications for prescribable medications were extracted using natural language processing and ontology relationships from six publicly available resources: RxNorm, Side Effect Resource 4.1, Mayo Clinic, WebMD, MedlinePlus, and Wikipedia. We compared the estimated precision and recall between the previous MEDI (MEDI-1) and the updated version (MEDI-2) with manual review. MEDI-2 contains 3031 medications and 186,064 indications. The MEDI-2 high precision subset (HPS) includes indications found within RxNorm or at least three other resources. MEDI-2 and MEDI-2 HPS contain 13% more medications and over triple the indications compared to MEDI-1 and MEDI-1 HPS, respectively. Manual review showed MEDI-2 achieves the same precision (0.60) with better recall (0.89 vs. 0.79) compared to MEDI-1. Likewise, MEDI-2 HPS had the same precision (0.92) and improved recall (0.65 vs. 0.55) than MEDI-1 HPS. The combination of MEDI-1 and MEDI-2 achieved a recall of 0.95. In updating MEDI, we present a more comprehensive medication-indication knowledgebase that can continue to facilitate applications and research with EHRs.

## Introduction

Medications and diagnoses are key components of clinical data. Linking medications and diagnoses can broadly facilitate clinical research such as evaluation of the quality of care and drug repurposing^1–5^. However, medications and diagnoses are recorded using different clinical terminologies in electronic health record (EHR), e.g., RxNorm and International Classification of Diseases (ICD). The lack of explicit medication-indication linkage between these terminologies hampers our ability to synthesize these valuable data for healthcare improvement. Indication and adverse effect are the two major relationships between medications and diagnoses: a medication’s intended treatment target is its indication, and an unexpected medical problem that happens during treatment with the medication is its adverse effect. Additionally, there are both on-label and off-label indications; on-label indications are those approved by the U.S. Food and Drug Administration (FDA) through clinical trials, whereas off-label indications are based on scientific evidence and collective physician experience after the FDA approval process^6^.

There have been several efforts to detail the relationships between medications and indications. Several resources, such as Side Effect Resource (SIDER), DrugBank, and LabeledIn, extracted indication from FDA’s structured product labels^7–10^. While these approach provides a robust source of on-label indications, these resources are unable to capture off-label indications^11^. Additionally, these resources only includes indications from currently FDA approved drug products^10^, which may leave out valuable information about no longer prescribable medications when researching with historical EHR data. Another resource, Chemical Entities of Biological Interests (ChEBI) includes indications in free unstructured text from Wikipedia, which is less readily applicable for EHR research than structured medication-indication relationships^12^. Some studies identified on-label and off-label indications by using frequently co-occurring medication-indication pairs in clinical notes^13,14^. However, differences across institutions, such as cohort demographics and provider diagnostic patterns, may affect the generalizability of these resources^15–18^. There are also several resources that focus primarily on adverse effects and not indications, including the “Large-scale Adverse Effects related to Treatment Evidence Standardization (LAERTES)” knowledgebase^19^.

In 2013, we introduced the publicly available MEDication-Indication (MEDI) knowledgebase that integrates information from four public medication resources (RxNorm, SIDER 2, MedlinePlus, and Wikipedia) to identify relationships between medications and their indications, including both on-label and off-label indications^20^. MEDI described medications with RxNorm concept unique identifiers (RxCUIs) and indications with ICD-9-CM (International Classification of Diseases, Ninth Revision, Clinical Modification) codes. Our original study demonstrated that the combination of resources could provide a more comprehensive coverage of indications than any single resource alone without compromising precision^20^. This observation was supported by the development of Drug Evidence Base (DEB), a medication indication knowledgebase which took a similar approach to aggregating both indication and adverse effect information from several resources^21^.

The first version of MEDI, which we will henceforth refer to as MEDI-1, has been well-utilized in pharmaceutical and clinical research^5,8,22–24^. However, the resource has become progressively outdated over the past seven years, limiting its usefulness^11^. For instance, some drugs are no longer commercially available in the U.S., and the FDA approved 220 novel drugs between 2015 and 2019^25^. The adoption of ICD Tenth Revision, Clinical Modification (ICD-10-CM) has also made MEDI-1 less applicable to modern EHRs. Furthermore, a number of the resources used to build MEDI-1 have been updated, such as SIDER 2, which has been updated to SIDER 4.1^7^.

In this paper, we present MEDI-2, an updated medication indication knowledgebase for biomedical research with modern EHRs. We built MEDI-2 with information from six public medication resources, including updated versions of the four original resources. With an updated manual review design, we evaluated the current release MEDI-2 and compared the precision and recall of MEDI-1, MEDI-2, and a combined MEDI-1 and MEDI-2 resource (MEDI-C).

## Results

### Summary of MEDI-2

**Figure 1:**
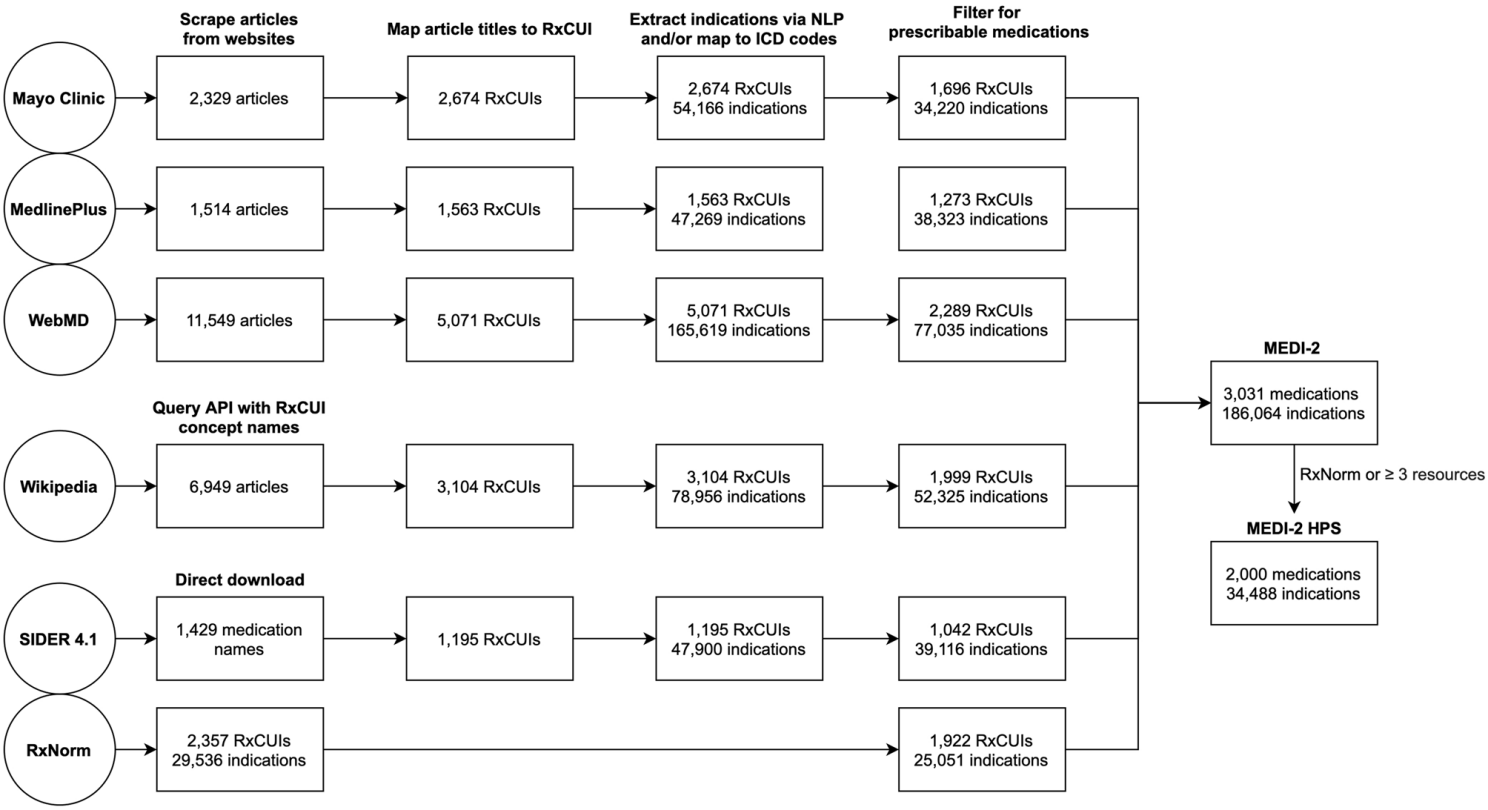
Flowchart outlining the process of building MEDI-2 and quantifying the medication and indications identified at each major step. RxCUI = RxNorm concept unique identifiers; HPS = high precision subset.

**Figure 2:**
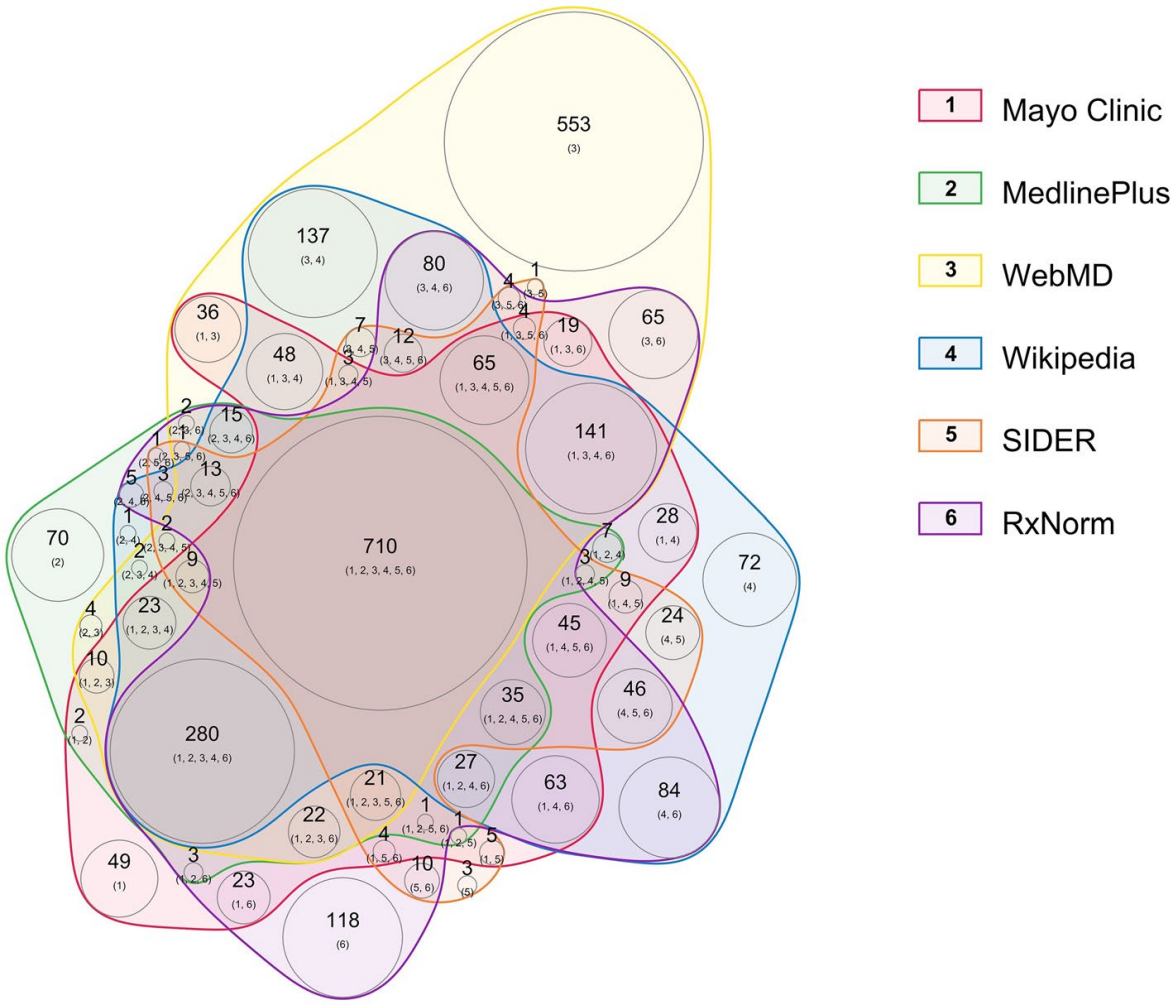
Weighted Venn diagram of distribution of medications within the six resources for MEDI-2. Each colored area represents a different resource. The larger number in each colored area represents the number of medications found in the combination of resources labeled by the smaller numbers in the parenthesis. The numbers in the parenthesis correspond with the numbers in the color legend. The circle area sizes are proportional to the number of medications–indications that were found within the corresponding resource(s).

A flowchart outlining the construction of MEDI-2 from the six resources is shown in Fig. 1. Briefly, medications from the resources were mapped to RxCUIs and indications were mapped first to United Medical Language System concept unique indentifiers (UMLS CUIs) and subsequently to ICD-9-CM and ICD-10-CM codes. The overlap between the six resources can be visualized in Fig. 2. Information was available from at least two resources for 2168 (71.6%) of these medications. Of note, 553 medications were found only in WebMD. However, many of these unique medications were traditional remedies, extracts, or oils such as coconut oil (RxCUI 1309239), Asian ginseng extract (RxCUI 1370774), or soy isoflavones (RxCUI 1807769).

**Figure 3:**
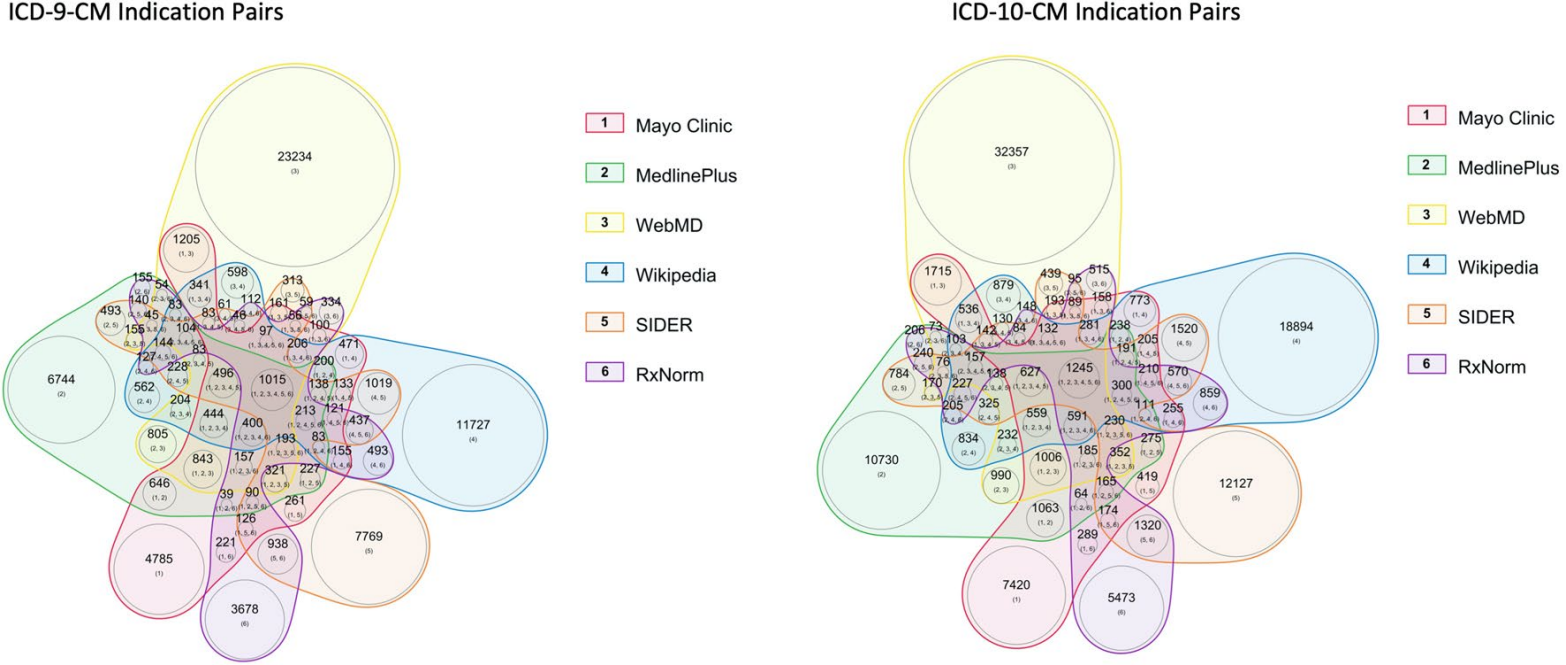
Weighted Venn diagram of distribution of medication-indication pairs within the six resources for MEDI-2, stratified by ICD-9-CM (left) and ICD-10-CM (right). Each colored area represents a different resource. The larger number in each colored area represents the number of medications found in the combination of resources labeled by the smaller numbers in the parenthesis. The numbers in the parenthesis correspond with the numbers in the color legend. The circle area sizes are proportional to the number of medications–indications that were found within the corresponding resource(s). *ICD* International Classification of Diseases.

Of the 3031 medications included in MEDI-2, we identified 4323 unique UMLS CUIs related to indications, giving 36,348 UMLS CUI medication-indication pairs. After mapping to ICD codes, there were 3072 unique ICD-9-CM codes and 5373 ICD-10-CM codes, resulting in 74,971 ICD-9-CM indications pairs and 111,093 ICD-10-CM indications pairs (Table 1). One medication can be paired with in an indication in both ICD-9-CM and ICD-10-CM. As shown in Fig. 3, a large proportion (77.8%) of these indication pairs were identified from only a single resource.

**Table 1:** Summary of counts of medications, ICD codes, and indications for MEDI-2.

Resource	Medications (% of total)	ICD-9-CM (% of total)	ICD-10-CM (% of total)	ICD-9-CM Indications (% of total)	ICD-10-CM Indications (% of total)
RxNorm	1922 (63.4)	1302 (42.4)	2125 (39.5)	10,221 (13.6)	14,830 (13.3)
Mayo Clinic	1696 (56.0)	1305 (42.5)	2137 (39.8)	14,027 (18.7)	20,193 (18.2)
MedlinePlus	1273 (42.0)	1270 (41.3)	2095 (39.0)	15,631 (20.8)	22,692 (20.4)
SIDER 4.1	1042 (34.4)	1833 (59.7)	3206 (59.7)	15,765 (21.0)	23,351 (21.0)
WebMD	2289 (75.5)	1484 (48.3)	2500 (46.5)	32,408 (43.2)	44,627 (40.2)
Wikipedia	1999 (66.0)	2417 (78.7)	4143 (77.1)	20,624 (27.5)	31,704 (28.5)
Union of all resources	3031	3072	5373	74,971	111,093

### Evaluation of MEDI-2

A summary of the estimated precision of different resource combinations is shown in Table 2. Medication-indication pairs extracted from the six resources had precisions ranging from 0.74 (Wikipedia) to 0.93 (RxNorm). While highly precise, RxNorm occasionally identifies false-positive indications when extracting indications from published abstracts of studies on cell lines or non-human subjects. For instance, RxNorm included the medication-indication pair amiloride and ‘disease of pancreas, unspecified’ (ICD10CM K86.9), where amiloride has been tested only in pancreatic cancer cell lines^26^.

**Table 2:** Estimated precision of MEDI-2 for different resource combinations.

Resource	Medications	Indications pairs	Total reviewed ^a^	True positive	Precision
RxNorm	1922	25,051	91	85	0.93
Mayo Clinic	1696	34,220	106	86	0.81
MedlinePlus	1273	38,323	105	82	0.78
SIDER 4.1	1042	39,116	102	78	0.76
WebMD	2289	77,035	126	95	0.75
Wikipedia	1999	52,325	111	82	0.74
**Excluding RxNorm**					
1 resource	2892	135,787	174	87	0.50
2 resources	1517	15,789	88	65	0.74
3 resources	939	5863	63	56	0.89
4 resources	510	2451	60	56	0.93
5 resources	233	1123	57	52	0.91
≥ 1 resource	2899	161,013			0.55
≥ 2 resources	1621	25,226			0.80
≥ 3 resources	1066	9437			0.90
≥ 4 resources	602	3574			0.92
MEDI-2 (any resource)	3031	186,064			0.60
MEDI-2 HPS ^**b**^	2000	34,488			0.92

^a^Indications that the reviewers deemed were too ambiguous were excluded from analysis (e.g., ICD10CM R69 = Illness, unspecified).

^b^HPS: High precision subset = indications from RxNorm or ≥ 3 resources.

Using a combination of resources excluding RxNorm, precision improved as we increased the threshold for the number of resources that contain the medication-indication pair. We selected the high-precision subset for MEDI-2 to include all medication-indication pairs from RxNorm or ≥ 3 resources. Therefore, MEDI-2 high precision subset (HPS) is composed of 2000 medications and 34,448 indication pairs, including both ICD-9-CM and ICD-10-CM indications.

### Comparison of MEDI-1, MEDI-2 and MEDI-C

In our original study, MEDI-1 included 3112 medications and 63,343 medication-indication pairs^20^. After regrouping MEDI-1 using the same generic ingredient groupings that we used for MEDI-2, there were 2701 unique medications and 56,550 medication-indication pairs remaining in MEDI-1. The decrease in number of medication and medication-indication pairs was likely due to changes in RxNorm relationships. For example, ‘morphine sulfate’ (RxCUI 30236) and ‘morphine hydrochloride’ (RxCUI 235751) in MEDI-1 were both mapped to ‘morphine’ (RxCUI 7052) in MEDI-2, reducing the number of medication-indication pairs from 33 to 20 for this drug.

**Figure 4:**
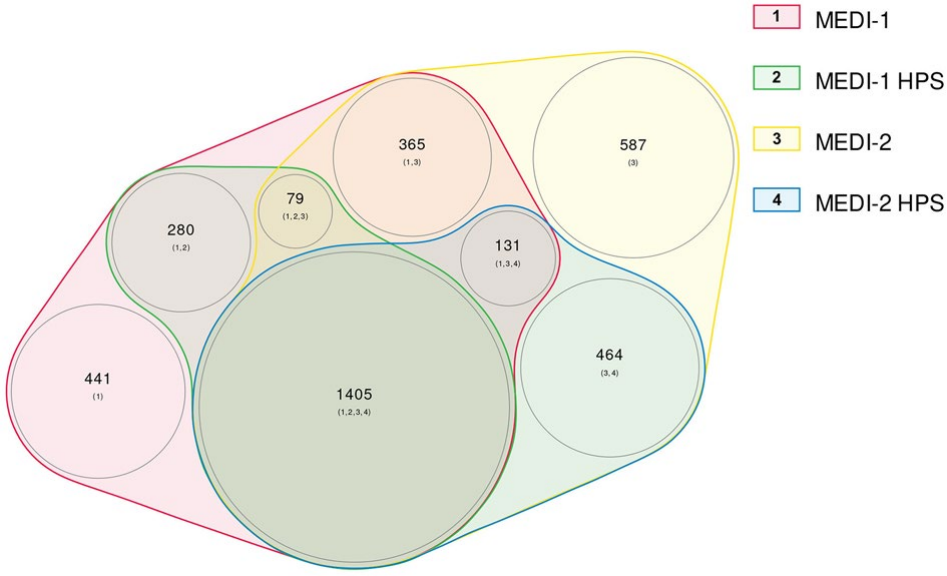
Weighted Venn diagram of the differences and overlap of medications included in MEDI-1, MEDI-1 HPS, MEDI-2, and MEDI-2 HPS. Each colored area represents a different resource. The larger number in each colored area represents the number of medications found in the combination of resources labeled by the smaller numbers in the parenthesis. The numbers in the parenthesis correspond with the numbers in the color legend. The circle area sizes are proportional to the number of medications found within the corresponding resource(s). *HPS* high precision subset.

A Venn diagram illustrating the overlap and differences between the medications included in MEDI-1 and MEDI-2 is shown in Fig. 4. There were 721 medications found only in MEDI-1 and not in MEDI-2, of which 254 (35.2%) are multi-ingredient medications, which we did not compare directly with MEDI-2 due to lack of standardization. Of the remaining 467 single-ingredient medications found only in MEDI-1, 79 medications were flagged by RxNorm as prescribable. In contrast, MEDI-2 has 1051 additional prescribable medications than MEDI-1, including 93 multi-ingredient medications and 652 (62.0%) prescribable single-ingredient medications that are unique to MEDI-2. Thus, for prescribable single-ingredient medications, MEDI-2 adds a significant number of medications (652) compared to MEDI-1, and only misses a small portion of medications (79) that were captured in MEDI-1.

For the high precision subsets, MEDI-1 HPS included 1764 medications and 11,552 indication pairs. There were 359 drugs identified in MEDI-1 HPS and not in MEDI-2 HPS, of which 152 (42.3%) are multi-ingredient medications. Within the 207 single-ingredient medications found only in MEDI-1 HPS, only 22 were prescribable. For MEDI-2 HPS, there are an additional 464 prescribable single-ingredient medications and 4 multi-ingredient medications.

In a review of 50 medication-indication pairs found in MEDI-1 HPS but not MEDI-2 HPS, we observed that 10 (20%) of the reviewed pairs were found to be invalid. Additionally, 32 (64%) of the reviewed pairs had related or better indications in MEDI-2 HPS. For example, although the medication-indication pair ‘albuterol’ (RxCUI 435) and ‘Acute bronchospasm’ (ICD-9-CM 519.11) was found only in MEDI-1, MEDI-2 identified similar indications for albuterol including ‘Acute bronchospasm’ (ICD-10-CM J98.01) and ‘Exercise induced bronchospasm’ (ICD-9-CM 493.81). Of the remaining 8 pairs that were valid only in MEDI-1 HPS, 5 of the 8 medications are not currently prescribable in the U.S., including cefamandole, chlorphenesin, streptokinase, pemoline, and valdecoxib. Therefore, we also grouped the indications from both MEDI-1 and MEDI-2 into MEDI-C since the combination will provide a higher recall for research with historical clinical data.

The estimated precision and recall for MEDI-1, MEDI-2, and MEDI-C (the combination of MEDI-1 and MEDI-2) is shown in Table 3. The reported number of indication pairs for MEDI-2 are markedly greater than MEDI-1 since it includes both ICD-9-CM and ICD-10-CM indications. Both MEDI-2 and MEDI-2 HPS have similar precision and improved recall compared to MEDI-1 and MEDI-1 HPS, respectively. MEDI-C, the combined version of MEDI-1 and MEDI-2, has a much higher recall (0.95) compared to MEDI-1 (0.79) and MEDI-2 (0.89) alone. Similarly, we observed that MEDI-C HPS has improved recall (0.67) compared to MEDI-1-HPS (0.55) and MEDI-2-HPS (0.65) alone. These observations suggest there are medications or indications identified in MEDI-1 that are not available in MEDI-2, likely because some medications have been commercially withdrawn from U.S. as observed in our reviews.

**Table 3:** Estimated precision and recall of MEDI-1 and MEDI-2.

Resource	Medications	Indications pairs ^a^	Precision ^b^	Recall
MEDI-1	2701	56,550	0.60	0.79
MEDI-1 HPS	1764	11,552	0.92	0.55
MEDI-2	3031	186,064	0.60	0.89
MEDI-2 HPS	2000	34,488	0.92	0.65
MEDI-C (MEDI-1 + MEDI-2)	3752	223,153	0.60	0.95
MEDI-C HPS (MEDI-1 HPS + MEDI-2 HPS)	2359	39,100	0.92	0.67

^a^Indication pairs for MEDI-2 include both ICD-9-CM indications and ICD-10-CM indications, which may include some overlap.

^b^Estimated precision for MEDI-1 and MEDI-1-HPS from Wei et al.^20^.

## Discussion

MEDI-2 is a comprehensive medication-indication knowledgebase prepared for biomedical research with modern EHRs. Leveraging information from six publicly available medication resources allows MEDI-2 to capture a broad range of medications and indications, improving precision and recall over any one resource alone^20^. Moreover, indications in MEDI-2 are represented with the widely-used ICD-9-CM and ICD-10-CM billing codes, allowing MEDI-2 to be easily utilized for research in many EHR systems.

Compared to MEDI-1, MEDI-2 captures many more medications and indications, and also modernizes MEDI by capturing ICD-10-CM indications. Despite the sharp increase in medication-indication pairs in MEDI-2, our review showed that MEDI-2 has an overall improved performance compared to MEDI-1, improving recall (0.89 vs. 0.79) without sacrificing precision. Similarly, MEDI-2 HPS also increased the overall number of covered medications (2000 vs. 1764) with improved recall (0.65 vs. 0.55) compared to MEDI-1 HPS.We also observed that the precision for individual resources was better in MEDI-2 compared to MEDI-1. For instance, the precision for Wikipedia improved from 0.56 in MEDI-1 to 0.74 in MEDI-2. This may be due to improvements in the resources themselves or in our pipeline for extracting and mapping indications.

Our review showed that a significant portion of medication-indication pairs (64%) found only in MEDI-1 HPS had a similar or better indication in MEDI-2 HPS and an additional 20% found only in MEDI-1 HPS were invalid pairs. Notably, the review identified five drugs that are no longer prescribable in the U.S., which may still be valuable when conducting research with longitudinal and historical EHR data. Therefore, we are also releasing MEDI-C, which will incorporate MEDI-1 into MEDI-2 with a flag to indicate which resource the medication-indication pair is from. Our reviews found that MEDI-C achieved a much higher recall of 0.95 than MEDI-1 (0.79) and MEDI-2 (0.89) alone.

A notable obstacle for MEDI-2 was the mapping of indications from free text in the articles to ICD. When rebuilding MEDI, we observed that the UMLS CUIs that were extracted by natural language processing (NLP) for indications did not always map to ICD codes. For instance, a free text mention of ‘breast cancer’ would be extracted by NLP as ‘Breast Carcinomas’ (CUI C067822), which only maps to Systematized Nomenclature of Medicine Clinical Terms (SNOMED CT) concepts for breast cancer through the UMLS. We were able to recapture some ICDs by mapping the SNOMED CT concepts to ICD, but it is possible some indications were overlooked. We also observed that ICD-10-CM indications were more easily captured than ICD-9-CM indication, which are still useful for research with older EHRs. This was likely due to updates in the UMLS concept tables that were used to map the UMLS CUIs extracted from the articles to ICDs. By integrating MEDI-1 into MEDI-2 for MEDI-C, we are able to keep more ICD-9-CM indications, but further work is needed to refine the mapping of free-text indications to ICD. Additionally, we provide the indications represented in UMLS CUIs alongside ICDs for MEDI-2, which can be useful for NLP tasks with free-text clinical notes.

Several limitations to MEDI-2 should be acknowledged. First, MEDI-2, like MEDI-1, is limited to medications and indications found in the public resources. Public resources are not perfect; we observed lower precision from indications extracted from < 3 resources. While most of the publicly available medication resources had significant overlaps with each other, there were 553 medications identified only in WebMD. As a consumer-based resource, WebMD often includes supplements and alternative/homeopathic medicines that were not found in the other resources. In particular, WebMD discusses many extracts or essential oils where the benefits may have limited evidence.

Concept extraction via NLP still remains a challenge with potential misrecognitions or mixing with adverse effects. MEDI-2 is primarily focused single-ingredient medications and likely excluded some prescription or branded medications that include a combination of medications. There is less naming standardization for multi-ingredient medications, which causes difficulty when mapping to RxCUIs. Additionally, the reported precisions and recalls in this study are imperfect estimates, but the lack of a gold standard makes it difficult to efficiently assess resources as large as MEDI without estimation from manual review. However, the similar precision calculated from manual reviews for both MEDI-1 and MEDI-2 supports our precision estimation. Lastly, MEDI only reports the binary relationships for medications-indications pairs and does not include more granular detail about the relationships for each pair, such as distinguishing between preventative or therapeutic indications. Additionally, we made no judgements on the strength of evidence for off-label indications. Indications mapped from SIDER 4.1, which is derived directly from the Food and Drug Administration’s structured product labels, may be considered as plausible evidence for ‘on-label’ indication^7^. Further work is needed to capture more detailed information in an automated manner.

In summary, MEDI-2 marks a significant improvement and expansion over our original medication-indication knowledgebase. Our results showed that incorporating new and updated resources enabled MEDI-2 to capture many additional medications and indications with greater recall. As a freely available and comprehensive resource, MEDI-2 can continue to enable in pharmaceutical and clinical research with EHRs.

## Methods

### Rebuilding MEDI with updated publicly available resources

We derived medication information from six publicly available resources: RxNorm, SIDER 4.1, Mayo Clinic, WebMD, MedlinePlus, and Wikipedia. Detailed descriptions of these resources are provided in Supplementary Table 1. RxNorm and SIDER 4.1 maintain medication-indication information in a structured table, while the other four resources are free-text based and are primarily focused on consumer health information.

For RxNorm, we retrieved all medication concepts and their associated RxCUIs from the prescribable subset of RxNorm^27^. Using the UMLS, we mapped the medications from RxNorm to indications represented by UMLS CUIs with the UMLS relationships ‘may_be_treated_by,’ ‘may_be_prevented_by,’ and ‘may_be_diagnosed_by. The ‘may_be_diagnosed_by’ relationship flag was included as it captured some true indications such as “levothyroxine” and “disorder of thyroid gland” or “papaverine” and “erectile disorder.” SIDER 4.1 provided medication names as free text and indications as UMLS CUIs^7^. We mapped the SIDER 4.1 medication names to RxCUIs by string matching with the UMLS.

Mayo Clinic, WebMD, and MedlinePlus all maintain directories of articles describing medications. We wrote a Python bot that automatically scraped the article titles and body text from these directories, excluding article subsections that were related to side effects or contraindications. We mapped the article titles to RxCUIs and combined articles with the same RxCUI. Articles that mapped to several RxCUIs contribute the same indications to each of the mapped medications. For Wikipedia, we extracted articles by querying Wikipedia’s application programming interface using the RxCUI concept names (i.e., medication name). We used KnowledgeMap Concept Indexer to identify medical concepts defined by UMLS CUIs in each medication document^28^. KnowledgeMap Concept Indexer is a locally developed NLP pipeline that has been shown to effectively extract medical concepts in medical documents and online resources^20,28,29^, outperforming the National Library of Medicine’s MetaMap NLP tool in precision and recall^28,30^. Medical concepts that were negated were excluded. We filtered the UMLS CUIs for the following semantic types: Disease or Syndrome, Congenital Abnormality, Acquired Abnormality, Anatomical Abnormality, Neoplastic Process, Virus.

The final version of MEDI-2 includes separate medication-UMLS CUI and medication-ICD code relationships. The identified UMLS CUIs from each resource were mapped to ICD-9-CM and ICD-10-CM codes with the UMLS concept tables. For CUIs that did not directly map into ICD but mapped to SNOMED-CT concepts, we used SNOMED-CT to ICD mappings from the National Library of Medicine (https://www.nlm.nih.gov/healthit/snomedct/archive.html; accessed January 2020). For instance, the UMLS does not map ‘Breast Carcinomas’ (CUI C067822) to ICD codes but does map to the SNOMED CT concepts for breast cancer. For UMLS CUIs that mapped to several ICD codes, each ICD code was considered as unique indications. Based on relationships within RxNorm, all medication concepts were grouped by their generic ingredient when possible (e.g. ‘tylenol’ is in group ‘acetaminophen’). Medications that included multiple active ingredients were mapped to a combined multi-ingredient generic when possible (i.e., ‘tylenol with codeine’ mapped to ‘acetaminophen / codeine) or to their single-ingredient components if not. We additionally regrouped MEDI-1 to generic ingredients using the same groupings for MEDI-2 for consistency.

### Evaluating MEDI-2

For MEDI-1, we demonstrated that combining multiple independent resources improved the precision of the medication-indication pairs^20,31^, We created a high-precision subset for MEDI-1 (MEDI-1 HPS) of medication-indication pairs that were either extracted from RxNorm, which already had high precision alone, or two or more other resources.

We estimated the precision of MEDI-2 to evaluate whether adding two additional resources would affect our threshold for the high-precision subset. First, an author with clinical background (NSZ) evaluated randomly selected subsets of 50 medication-indication pairs from each of the six resources used to build MEDI-2. The positive predictive value (PPV) for each resource was calculated by dividing the number of true positive medication-indication pairs by the total number of pairs reviewed. Reviewed medication-indication pairs that were found in more than one resource were included in the respective PPV calculation for each of the overlapping resources. Then, excluding RxNorm, we had two authors with a clinical background (VB and HNE) evaluate additional subsets of 50 medication-indication pairs derived from two, three, four, and five resources, respectively. Medication-indication pairs were deemed ‘true’ if the reviewers found evidence for the indication in UpToDate, clinical trials, or peer-review studies. Ambiguous indications, namely ICD codes that are too broad (e.g., ICD-10-CM R69 = ‘Illness, unspecified’), were excluded from analysis. The reviewers used studies published in peer-reviewed journals and UpToDate (https://www.uptodate.com), an evidenced-based clinical resource commonly used by practicing clinicians, in their evaluation.

We estimated the precision of a combination of resources (R) with the following equation:

$$Precision\left(R\right)=\frac{{\sum }_{r\in R}size\left(r\right)\times PPV\left(r\right)}{{\sum }_{r\in R}size\left(r\right)}$$

where $R$ is the set (combination) of reviewed resources $r$, $size(r)$ is the number of medication-indication pairs in resource $r$, and $PPV(r)$ is the estimated positive predictive value for resource $r$ from the reviews.

### Comparison of MEDI-1, MEDI-2, and MEDI-C

We estimated the precision of MEDI-1, MEDI-2, and MEDI-C (the combination of MEDI-1 and MEDI-2) using the above-defined precision equation. A board-certified physician clinician (VEK) also reviewed 50 medication-indication pairs that were found in MEDI-1 HPS, but not in MEDI-2 HPS. For each medication-indication pair in the review subset, the clinician also indicated whether a similar or better indication was in MEDI-2 HPS.

In this update, we also designed an experiment to estimate the recall. We pre-selected five common medications that have multiple indications which span several domains: propranolol, methotrexate, sildenafil, gabapentin, and estradiol. A physician with board-certification in internal medicine (VEK) used UpToDate to curate a list of clinically-accepted on-label and off-label indications for the five medications. Then, the clinician reviewed the medication-indication pairs for the five medications from the three resources and indicated whether indications from their initial list were found in each resource.

## Supplementary Information

Supplementary Information.

## Data Availability

MEDI is made freely available for download at https://www.vumc.org/wei-lab/medi. Code and scripts used to construct MEDI are made available upon request (wei-qi.wei@vumc.org).
