# Supplementary material for: An updated, computable MEDication-Indication resource for biomedical research
Source: Sci Rep. 2021 Sep 23;11:18953. doi: 10.1038/s41598-021-98579-4 (PMC8460636; doi:10.1038/s41598-021-98579-4)
Supplement: Supplementary file 1 — Supplementary Information. [file 41598_2021_98579_MOESM1_ESM.docx]

**An updated, computable MEDication-Indication resource for biomedical research**

Neil S. Zheng, V. Eric Kerchberger, Victor A. Borza, H. Nur Eken, Joshua C. Smith, Wei-Qi Wei

| **Supplementary Table 1. Description of online medical resources used to construct MEDI-2** | | |
| --- | --- | --- |
| **Resource Names** ^a^ | **Descriptions** | **Links** |
| RxNorm | an ontology for standardizing medication information among clinical systems | <https://www.nlm.nih.gov/research/umls/rxnorm/index.html> |
| Side Effect Resource  (SIDER) 4.1 | a resource of indications and adverse drug reactions extracted from the U.S. Food and Drug Administration’s structured product labels | <http://sideeffects.embl.de/> |
| Mayo Clinic | a consumer-focused medical information website operated by Mayo Clinic | <https://www.mayoclinic.org> |
| MedlinePlus | a medical encyclopedia maintained by the National Library of Medicine | [https://medlineplus.gov/ encyclopedia.html](https://medlineplus.gov/encyclopedia.html) |
| WebMD | a consumer-focused medical information website | <https://www.webmd.com/> |
| Wikipedia | a collaboratively edited encyclopedia | <https://www.wikipedia.org/> |
| ^a^ All resources accessed in January 2020 | | |
